# Supplementary material for: Scenario-based forecast of the evolution of 75 years of unrest at Campi Flegrei caldera (Italy)
Source: Commun Earth Environ. 2026 Jan 12;7(1):37. doi: 10.1038/s43247-025-03140-0 (PMC12795757; doi:10.1038/s43247-025-03140-0)
Supplement: Supplementary file 2 — Supplementari Information [file 43247_2025_3140_MOESM2_ESM.pdf]

SUPPLEMENTARY INFORMATION

Scenario-based forecast of the evolution of 75 years of unrest at Campi Flegrei caldera (Italy)

Luca Caricchi[1*], Charline Lormand[1], Stefano Carlino[2], Tommaso Pivetta[2], Guy Simpson[1]

1) Department of Earth Sciences, University of Geneva, Geneva, Switzerland
2) INGV-Sezione di Napoli, Osservatorio Vesuviano, Via Diocleziano 328, 80124 Napoli, Italy

*Corresponding author Luca Caricchi: luca.caricchi@unige.ch

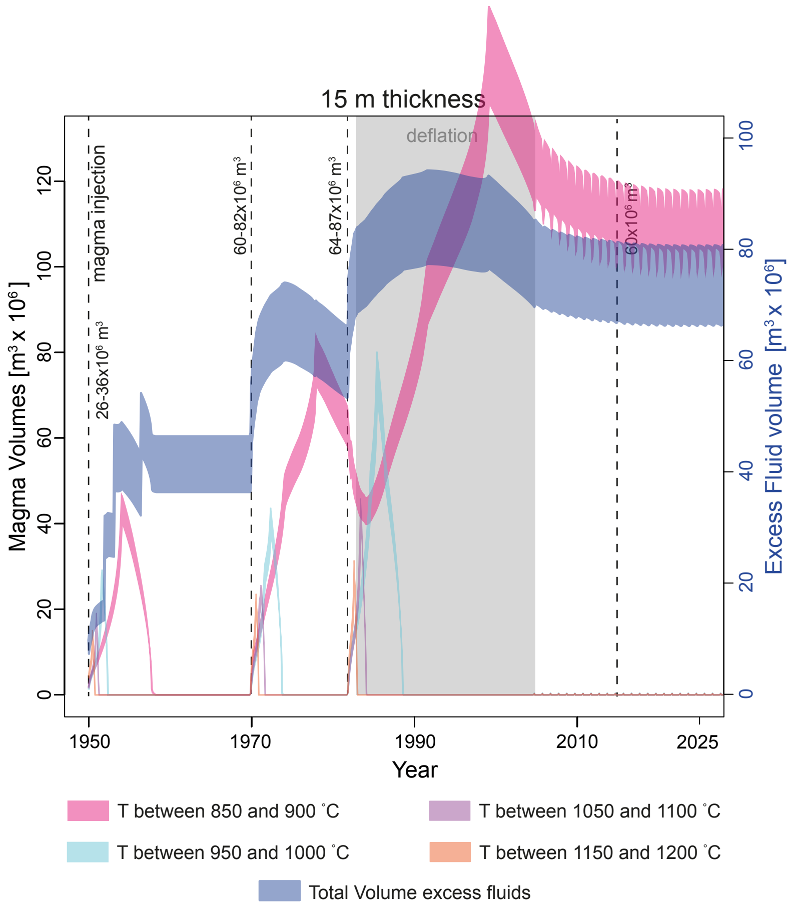

***Figure S1: Temporal evolution of the volume of magma at different temperatures and excess fluids**. The figure reports the results for the injections of 15m thickness, and the maximum and minimum volume of the magma injection events (Table S3). The lower end of each coloured region is for the calculations performed considering the minimum volume of injected magma at each event. The numbers close to the dashed lines indicate the minimum*

and maximum volume of magma injected at each episode. The regions of different colour represent the volumes of excess fluids (in blue), and magma within different temperatures present within the magma reservoir over time.

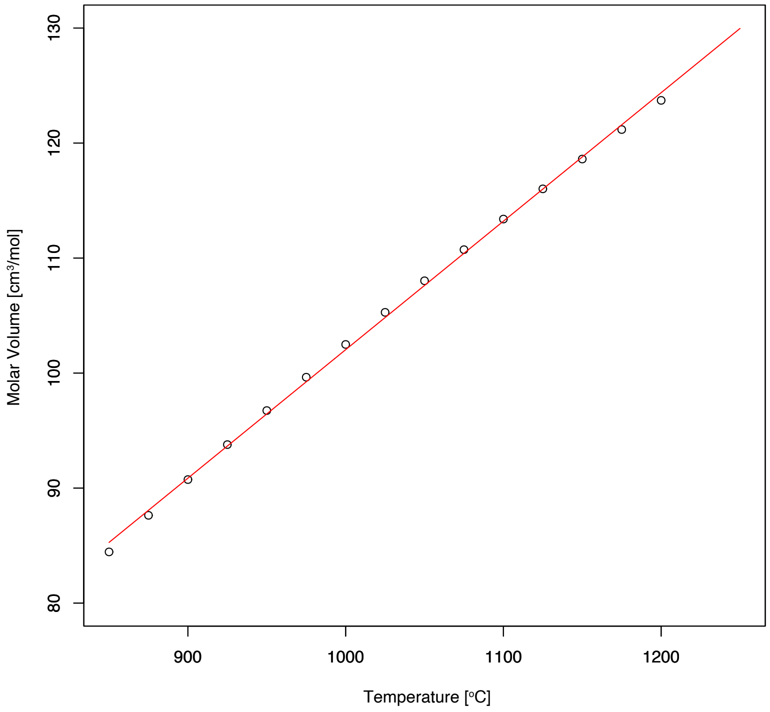

Figure S2: Molar volume of H_2O as function of temperature at 100 MPa (Ref.* [47]*). *The red line shows the regression that was used to compute the molar volume of excess fluids from the thermal modelling results.*

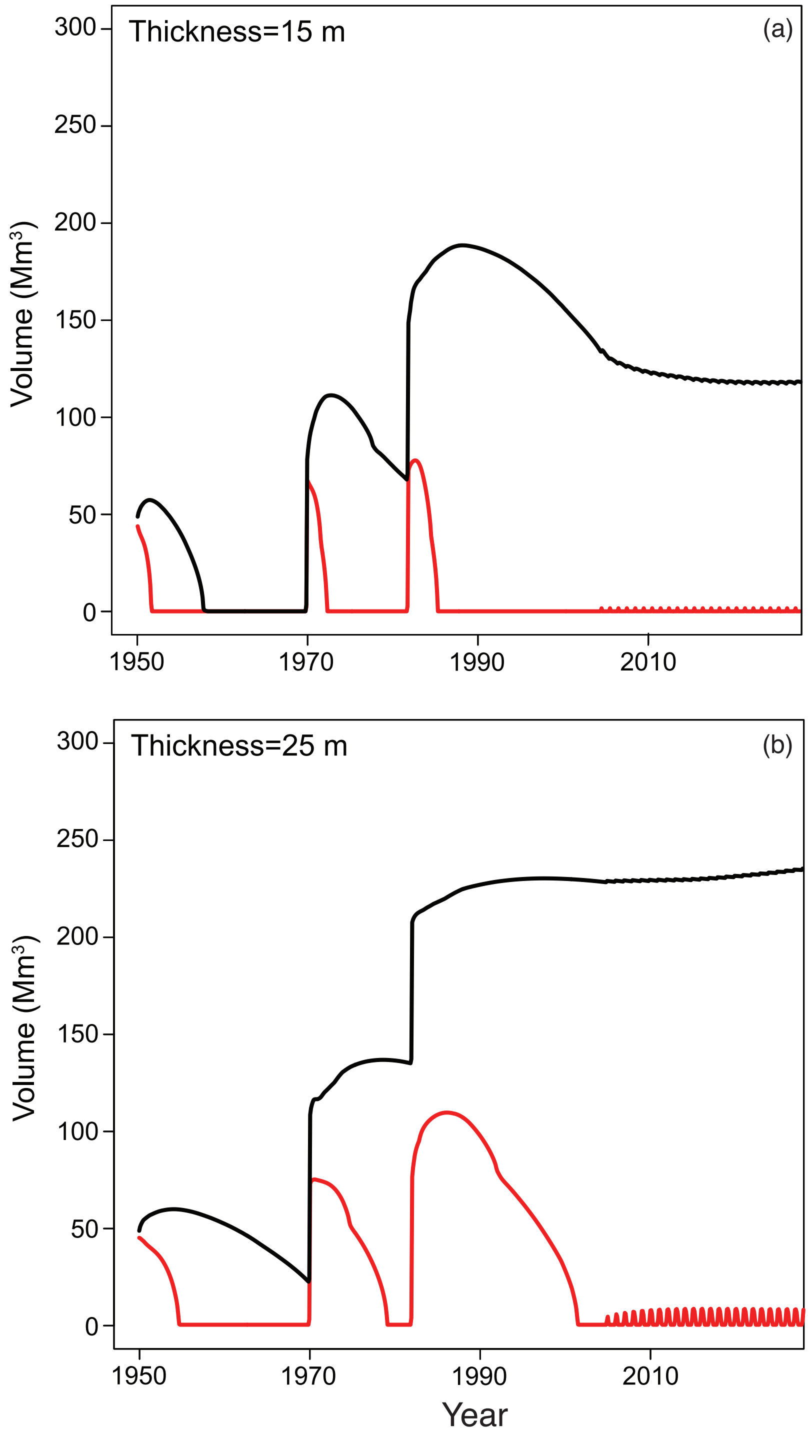

Figure S3: Temporal evolution of the reservoir volume in black and volume of eruptible magma in red*. The results are for the maximum volumes of the injections for sills of 15m (a) and 25m thickness (b) as reported in Table S2.*

Table S1: Estimates of volumetric variations associated with the 4 unrest episodes, and diameters of the sill used for thermal modelling

Unrest period	ΔV $m^3 \times 10^6$	Sill diameter (m) for h=15 m	Sill diameter (m) for h=25 m
1950-1952	26 - 36	742 - 874	575 - 677
1970-1972	60 - 82	1128 - 1319	874 - 1021
1982-1984	64 - 87	1165 - 1359	902 - 1052
2007-2023	60	1128	874

Table S2: Calculated current volume of the magma reservoir, eruptible volume, and volume fraction of excess fluids that would be generated by the injection at the current inverted rates of 8 x 10^6 m^3/y for different model configurations

Thickness of injections (m)	Vol injections (km^3) x 10^{-2}	Vol. reservoir (km^3)	Vol. eruptible (km^3)	Vol. Fraction exsolved fluids (closed system)
15	26 - 60 - 64 - 60	0.10	0.002	0.28
15	36 - 82 - 87 - 60	0.11	0.002	0.28
25	26 - 60 - 64 - 60	0.20	0.06	0.23
25	36 - 82 - 87 - 60	0.23	0.08	0.23

Table S3: Parameters used for thermal modelling

Parameter	Unit	Value
Geotherm	°C.m^{-1}	180
Intrusion temperature	°C	1250
Solidus temperature	°C	850
Liquidus temperature	°C	1250
Time step	years	0.1
Density	kg.m^{-3}	2500
Latent heat	J.kg^{-1}	350000
Specific heat capacity	J.kg^{-1}.K^{-1}	1000
