# Supplementary material for: Scenario-based forecast of the evolution of 75 years of unrest at Campi Flegrei caldera (Italy)
Source: Commun Earth Environ. 2026 Jan 12;7(1):37. doi: 10.1038/s43247-025-03140-0 (PMC12795757; doi:10.1038/s43247-025-03140-0)
Supplement: Supplementary file 3 — Description of Additional Supplementary File [file 43247_2025_3140_MOESM3_ESM.pdf]

Description of Additional Supplementary Files

File name: Supplementary Data 1

Description: Calculated current volume of the magma reservoir, eruptible volume, and volume fraction of excess fluids that would be generated by the injection at the current inverted rates of 8 x 106 m3/y for different model configurations
